# An Inflammatory Fibroid Polyp of the Stomach

**DOI:** 10.7759/cureus.62001

**Published:** 2024-06-09

**Authors:** Rumyana Krasteva, Silvia Ivanova, Magdalena Alexieva, Mila Kovacheva-Slavova, Borislav Vladimirov, Georgi Yankov

**Affiliations:** 1 Pathology, University Hospital “St. Ivan Rilski”, Medical University of Sofia, Sofia, BGR; 2 Thoracic Surgery, University Hospital “St. Ivan Rilski”, Medical University of Sofia, Sofia, BGR; 3 Gastroenterology, University Hospital “Tsaritsa Ioanna-ISUL”, Medical University of Sofia, Sofia, BGR

**Keywords:** vaněk’s polyp, fibroid polyp, gastrectomy, stomach, inflammatory fibroid polyp

## Abstract

An inflammatory fibroid polyp (Vaněk’s polyp) is a rare, benign, mesenchymal polyp originating from the submucosa of the gastrointestinal tract. Symptoms are non-specific and depend on the tumor size and location. Despite their benign nature, these tumors can mimic other malignant conditions, making an accurate diagnosis crucial for appropriate management. Histologically, they are submucosal lesions composed of spindle-shaped or stellate stromal cells, stroma with thin-walled vessels around which spindle-shaped cells are arranged similar to onion skin, an eosinophil-rich inflammatory infiltrate, and minimal mitotic activity. In this article, we present the case of a 63-year-old woman with a giant benign inflammatory fibroid polyp of the stomach. We performed distal esophageal resection, total gastrectomy, and omentectomy, as the passage was restored with a transmesocolic termino-lateral esophago-jejunal Roux-en-Y anastomosis. We also present a brief literature review on this topic.

## Introduction

An inflammatory fibroid polyp (IFP) (Vaněk’s polyp) is a rare, benign, mesenchymal polyp originally described as an eosinophilic submucosal granuloma by Vaněk in 1949 [1]. IFP is also known as eosinophilic granuloma, granuloblastoma or gastric fibroma with eosinophilic infiltration, and inflammatory pseudotumor [2,3]. Although the etiology of IFPs remains unclear, the presence of eosinophils suggests an allergic reaction as other factors such as neural hyperplasia, irritants, trauma, genetic alterations, and bacterial, physical, or chemical stimulants have been implicated [4]. The stomach is the most commonly affected gastrointestinal organ [2]. IFPs represent fewer than 0.1% of all gastric polyps [5]. Women in their fifth decade are most commonly affected [2]. Symptoms are non-specific and depend on the tumor size and location. Despite their benign nature, IFPs can mimic other malignant conditions, making an accurate diagnosis crucial for appropriate management [6-9]. Typical endoscopic findings are solitary intraluminal masses or intramural sessile lesions [10]. Differentiating between gastric intramural tumors may be difficult because of overlapping appearances, and it is important to assess the combination of features, such as tumor location, margin, growth pattern, attenuation, and enhancement, to suggest a diagnosis [11,12]. On immunohistology, IFPs appear to be positive for CD34 and vimentin and negative for CD117 [13]. The differential diagnoses based only on histological findings include sarcoma and other malignancies as the absence of mutational change may help exclude malignant lesions [14].

## Case presentation

**Figure 1:**
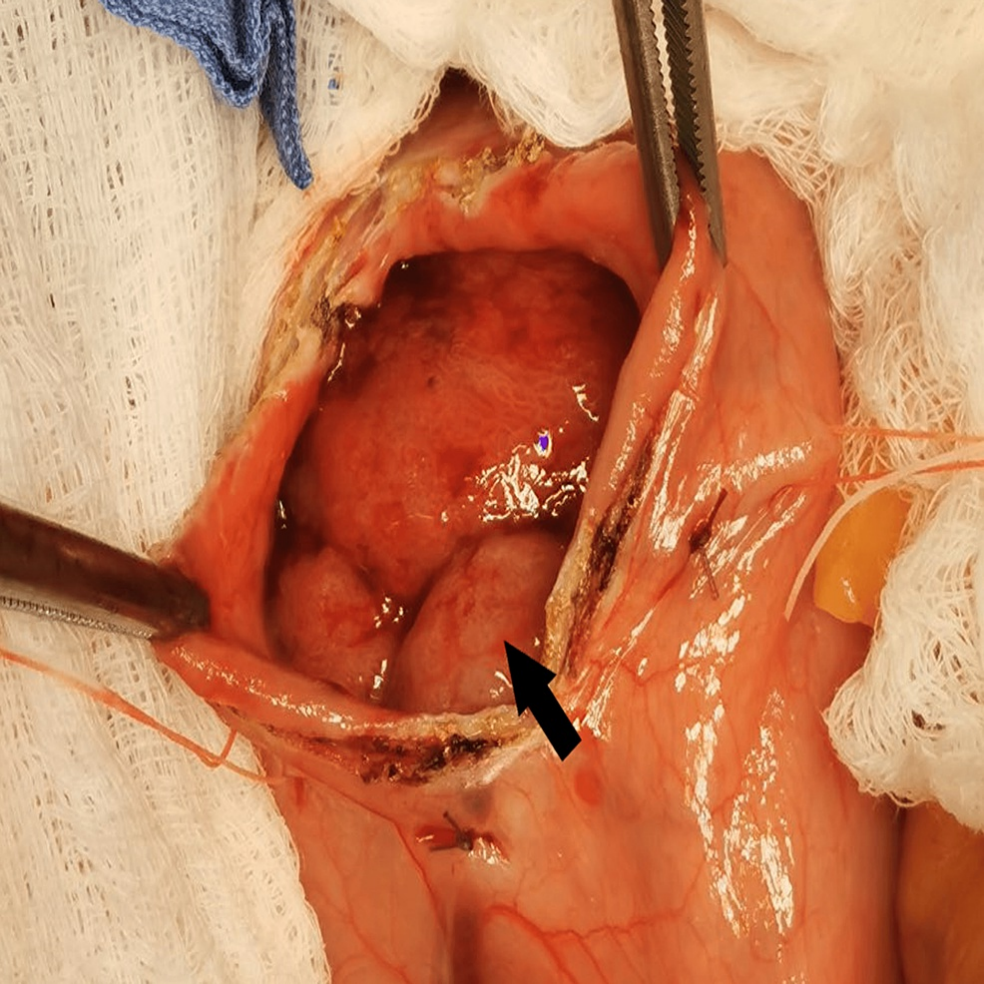
Intraoperative image of gastrotomy and a probable inflammatory fibroid polyp of the stomach.

A 63-year-old woman was admitted due to complaints of abdominal pain, fatigue, loss of appetite, and weight loss (about 5 kg) over seven months. Two upper endoscopies confirmed the presence of a large polypoid tumor with a diameter of approximately 90 mm and a lobulated easily bleeding surface located in the posterior wall of the gastroesophageal junction. Computed tomography (CT) showed a homogeneously hypodense lesion in the lumen of the stomach with axial dimensions of 70/100 mm and moderate thickening of the stomach wall in the area of the gastroesophageal junction up to 12 mm. The patient was referred for surgical treatment. An upper midline laparotomy was performed and a dense elastic tumor in the proximal portion of the stomach was palpated. We found a small axial hiatal hernia, traction, and acquired shortening of the abdominal esophagus, possibly due to the pre-existing gastroesophageal reflux disease. A vertical gastrotomy was performed, and a lobulated tumor measuring approximately 90/70 mm was found arising broadly from the posterior gastric wall just below the gastroesophageal junction (Figure 1). The latter was biopsied and sent for fresh frozen section analysis which was suggestive of a probable gastrointestinal stromal tumor (GIST). A left lateral mini-thoracotomy followed because of the marked shortening of the esophagus and the need to reach a proximal intact esophageal wall. Distal esophageal resection, total gastrectomy, and omentectomy were performed along with the removal of visibly thickened and borderline paraesophageal lymph nodes and slightly thickened and enlarged lymph nodes around the celiac trunk, left gastric artery, and splenic artery. A small bowel loop was brought approximately 40 cm distal to the ligament of Treitz in the left hemithorax, and the passage was restored with a transmesocolic termino-lateral esophago-jejunal Roux-en-Y anastomosis.

**Figure 2:**
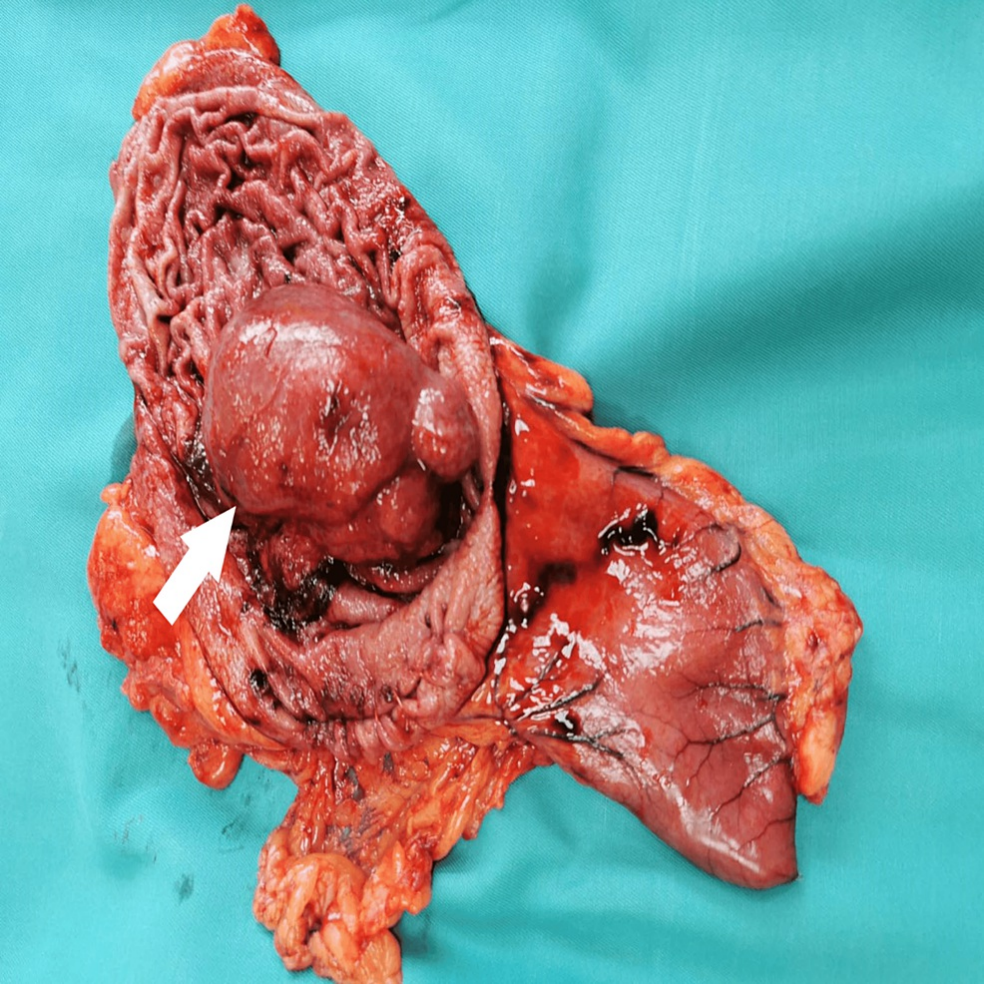
Postoperative specimen of the resected and cut stomach and an inflammatory fibroid polyp originating from it.

**Figure 3:**
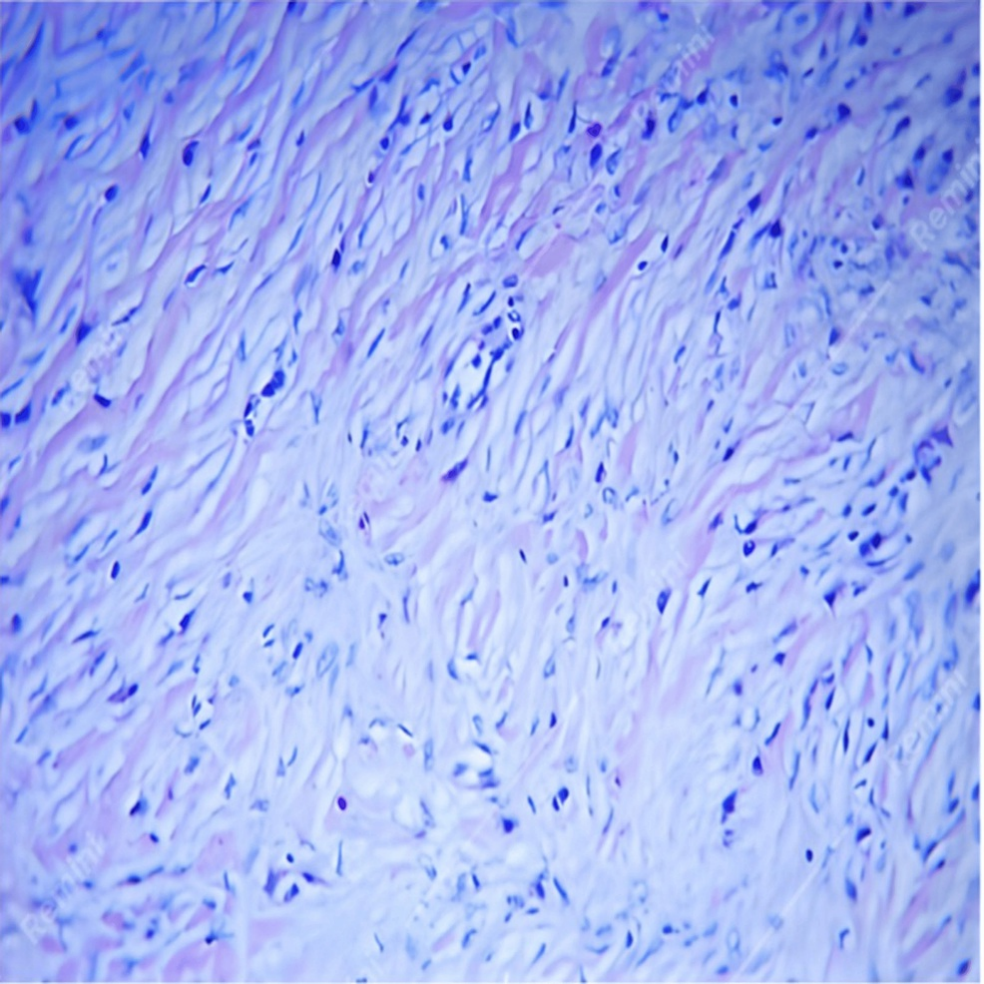
Microscopic image after hematoxylin-eosin staining of a gastric inflammatory fibroid polyp.

**Figure 4:**
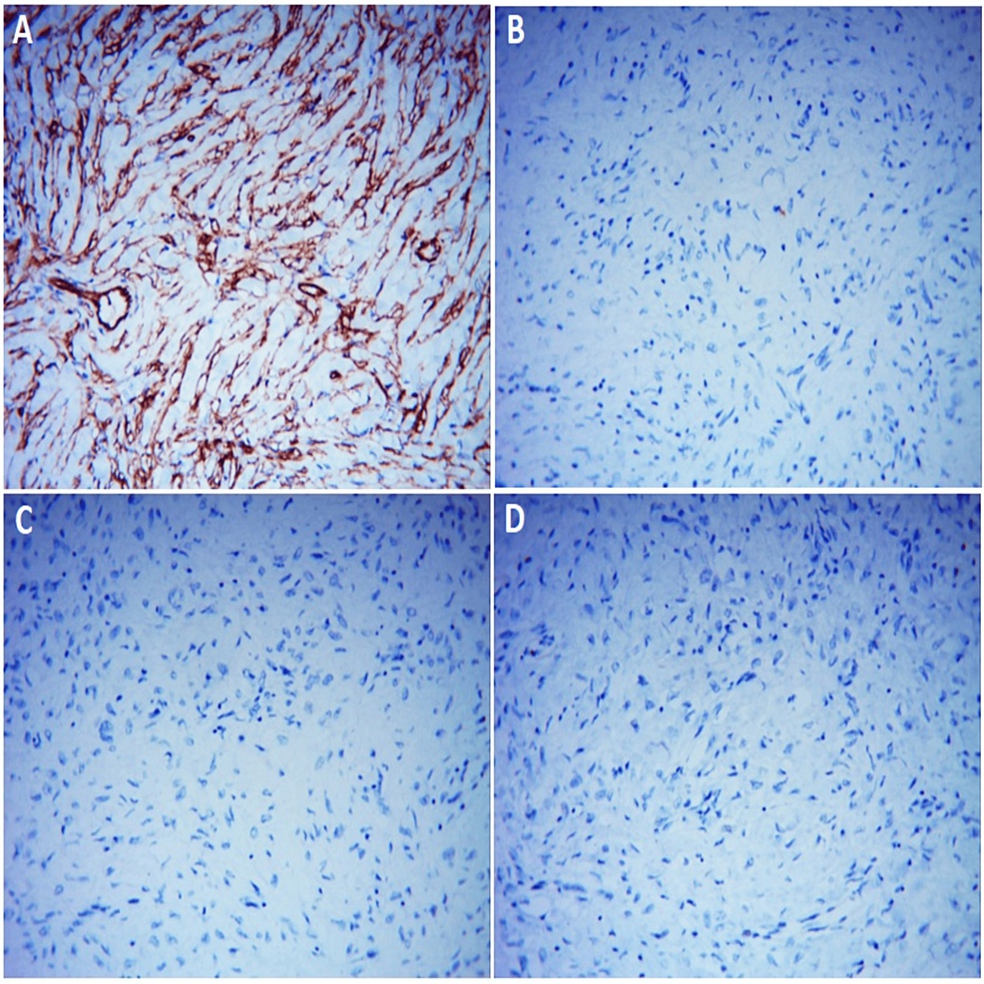
Microscopic images of immunohistochemistry of gastric inflammatory fibroid polyp showing (A) diffuse stromal expression of CD34, (B) negative stromal expression and positive expression in scattered mast cells of CD117, and (C and D) negative stromal expression of S-100 and DOG1.

On macroscopic examination of the resected material from the stomach, a rounded tumor growing on a broad base toward the lumen was observed. It was well-circumscribed and measured 90 × 65 × 70 mm (Figure 2). Microscopic histopathological evaluation showed the presence of a submucosal lesion represented by spindle-shaped to stellate stromal cells, loose edematous stroma with thin-walled blood vessels around which spindle-shaped cells were arranged like onion skins, and an inflammatory infiltrate rich in eosinophils and minimal mitotic activity (Figure 3). The dissected 20 lymph nodes had a preserved histological structure and the resection margins were negative. Immunohistochemical examination of CD117 showed negative stromal expression and positive expression in scattered mast cells. There was a diffuse stromal expression of CD34 and a negative stromal expression of S-100 and DOG1 (Figure 4). These findings excluded GIST, plexiform fibromyxoma, and schwannoma, and confirmed the diagnosis of a giant benign IFP of the stomach arising from the submucosa of the gastric wall.

## Discussion

IFP is a rare, benign, submucosal mesenchymal polyp of the gastrointestinal tract [1]. Although there is an association with an activating mutation in the gene platelet-derived growth factor receptor alpha (*PDGFRA*), the pathogenesis remains unclear [7].

They occur most often in the stomach (mainly in the antrum), but are also observed in the esophagus, small intestine, large intestine, and anal canal [6]. They are usually asymptomatic and are often an incidental finding during endoscopic examination. Symptomatic cases present with abdominal pain, weight loss, intestinal obstruction, ulcer-like symptoms, gastrointestinal bleeding, and iron deficiency anemia [8]. Various imaging modalities, including endoscopy, CT scans, and MRI, play a pivotal role in diagnosing IFPs. Definitive confirmation typically requires histopathological examination of the excised tissue. Histologically, IFPs are submucosal lesions composed of spindle-shaped or stellate stromal cells, stroma with thin-walled vessels around which spindle-shaped cells are arranged like onion skins, an eosinophil-rich inflammatory infiltrate, and minimal mitotic activity. Because of the deeper submucosal location of the characteristic spindle cells, this characteristic finding is rarely detected on endoscopic examination [7]. They show positive immunoreactivity for CD34, variable activity for actin, and negative activity for CD117, S-100 (c-kit), factor VIII, desmin, and cytokeratin [6,11]. Differential diagnosis is made with other intramural gastric tumors some of which have a characteristic histologic appearance, but it is often imperative to use immunohistochemistry to confirm the diagnosis. For example, c-kit (CD117) or DOG1 is present in GISTs; desmin in leiomyomas; S-100 in schwannomas; smooth muscle actin in glomus tumors; anaplastic lymphoma kinase in inflammatory myofibroblastic tumors; and chromogranin A and synaptophysin in carcinoids [11,12]. A large IFP is an indication for surgical intervention. In our case, because of the size of the lesion, its localization in the subcardiac part of the stomach, its proximity to the gastroesophageal junction, and the suspicion of GIST from the frozen section, it was necessary to perform a resection of the distal part of the esophagus, total gastrectomy, omentectomy, and left-sided intrathoracic esophago-jejuno-anastomosis by Roux en-Y. We decided to operate with a combined approach (laparotomy + lateral mini-thoracotomy) because of the concomitant small axial hiatal hernia and acquired esophageal shortening. Another possible option could be gastrectomy and transhiatal resection of the distal part of the esophagus with mechanical stapler suturing high in the mediastinum. We preferred the combined approach because of the safer performance of the anastomosis and removal of the paraesophageal lymph nodes. There are also laparoscopic techniques for gastrectomy, but given the unclear preoperative histology and the size of the tumor, in our opinion, they were not suitable in this case.

## Conclusions

Vaněk’s polyp of the stomach remains one of the rarest benign tumors of the gastrointestinal tract and presents a medical challenge from the diagnostics through the treatment of choice. Despite their benign nature, IFPs can mimic other malignant conditions, making an accurate diagnosis crucial for appropriate management. A multidisciplinary approach is needed. A definitive diagnosis is made histopathologically. Surgery is generally recommended for symptomatic patients and bleeding lesions with excellent long-term prognosis. More knowledge is necessary for this rare gastric tumor to improve the medical management of the patients.
